# A pneumatically powered knee-ankle-foot orthosis (KAFO) with myoelectric activation and inhibition

**DOI:** 10.1186/1743-0003-6-23

**Published:** 2009-06-23

**Authors:** Gregory S Sawicki, Daniel P Ferris

**Affiliations:** 1 Human Neuromechanics Laboratory, School of Kinesiology, University of Michigan, 401 Washtenaw Avenue, Ann Arbor, Michigan, 48109-2214, USA; 2 Department of Mechanical Engineering, University of Michigan, Ann Arbor, Michigan, USA; 3 Department of Biomedical Engineering, University of Michigan, Ann Arbor, Michigan, USA; 4 Department of Physical Medicine and Rehabilitation, University of Michigan, Michigan, Ann Arbor, USA

## Abstract

**Background:**

The goal of this study was to test the mechanical performance of a prototype knee-ankle-foot orthosis (KAFO) powered by artificial pneumatic muscles during human walking. We had previously built a powered ankle-foot orthosis (AFO) and used it effectively in studies on human motor adaptation, locomotion energetics, and gait rehabilitation. Extending the previous AFO to a KAFO presented additional challenges related to the force-length properties of the artificial pneumatic muscles and the presence of multiple antagonistic artificial pneumatic muscle pairs.

**Methods:**

Three healthy males were fitted with custom KAFOs equipped with artificial pneumatic muscles to power ankle plantar flexion/dorsiflexion and knee extension/flexion. Subjects walked over ground at 1.25 m/s under four conditions without extensive practice: 1) without wearing the orthosis, 2) wearing the orthosis with artificial muscles turned off, 3) wearing the orthosis activated under direct proportional myoelectric control, and 4) wearing the orthosis activated under proportional myoelectric control with flexor inhibition produced by leg extensor muscle activation. We collected joint kinematics, ground reaction forces, electromyography, and orthosis kinetics.

**Results:**

The KAFO produced ~22%–33% of the peak knee flexor moment, ~15%–33% of the peak extensor moment, ~42%–46% of the peak plantar flexor moment, and ~83%–129% of the peak dorsiflexor moment during normal walking. With flexor inhibition produced by leg extensor muscle activation, ankle (Pearson r-value = 0.74 ± 0.04) and knee ( r = 0.95 ± 0.04) joint kinematic profiles were more similar to the without orthosis condition compared to when there was no flexor inhibition (r = 0.49 ± 0.13 for ankle, p = 0.05, and r = 0.90 ± 0.03 for knee, p = 0.17).

**Conclusion:**

The proportional myoelectric control with flexor inhibition allowed for a more normal gait than direct proportional myoelectric control. The current orthosis design provided knee torques smaller than the ankle torques due to the trade-off in torque and range of motion that occurs with artificial pneumatic muscles. Future KAFO designs could incorporate cams, gears, or different actuators to transmit greater torque to the knee.

## Background

Powered lower-limb orthoses (i.e. robotic exoskeletons) can be useful tools for assisting gait rehabilitation therapy and studying the neuromechanics and energetics of human locomotion [1-3]. A primary goal of these devices is to replace or restore a portion of the torque and/or mechanical work performed by the biological muscle-tendons acting at the joints (e.g. ankle, knee or hip) during locomotion. Ideally, the mechanical assistance is delivered while maintaining overall kinetic and kinematic patterns similar to normal walking so that they provide little disruption to gait.

In our previous research, we built and tested lightweight carbon-fiber ankle-foot orthoses (AFO) with artificial pneumatic muscles capable of powering both ankle plantar flexion and dorsiflexion during human walking [1,4,5]. We concentrated our initial efforts on the ankle because it plays a crucial functional role during normal walking. The healthy plantar flexors (e.g. soleus, gastrocnemius) aid in (1) forward propulsion (2) swing initiation and (3) body-weight support [6-8] during walking. The plantar flexors are a major source of mechanical energy, contributing 35%–50% of the total positive mechanical work over a stride [9-11]. Most of this work is performed at push-off, when ankle muscle-tendons help drive the step-to-step transition, propelling the body upward and forward to maintain steady walking speed [12].

Muscle-tendons spanning the knee also greatly influence normal walking dynamics and should be considered in the design of assistive devices. Healthy knee extensors and flexors act both to absorb and generate energy at different phases over the walking stride. During initial stance, the knee joint extensors prevent the leg from buckling: acting to support body-weight while performing negative mechanical work (e.g. similar to a shock absorber). During mid- and late stance, the knee generates mechanical energy, some of which may be recycled energy stored previously in elastic tissues during the absorption phase [12]. At the stance-swing transiton, the knee muscle-tendons stabilize the limb during push-off and then absorb energy to control leg motion during swing.

Several powered orthoses have been tested to aid the knee during human walking. Some of the designs provided real-time mechanical assistance using quasi-passive magnetorheological variable dampers [13], linear hydraulic actuators [14], electric actuators [15-18] and variable stiffness actuator springs [19]. Artificial pneumatic muscles have recently been tested on a powered hip orthosis [20], but we are unaware of any device that has used artificial pneumatic muscles to provide torque assistance at the knee. In addition, perhaps because of added hardware and software design complexity, few devices have been described that can simultaneously provide active torque to drive both ankle plantar/dorsiflexion and knee flexion/extension. It is difficult to evaluate the performance of most of the prototypes because gait analysis data from users walking in them is limited [21].

The overall goal of this study was to extend our pneumatically powered ankle orthosis concept to the knee, and test its performance on healthy human walkers. We built a unilateral powered knee-ankle-foot orthosis (KAFO) with antagonistic pairs of artificial pneumatic muscles at both the ankle (i.e. plantar flexor and dorsiflexor) and the knee (i.e. extensors and flexors). The orthosis pneumatic muscles were controlled using surface electromyography recordings from the user's own biological muscles (i.e. proportional myoelectric control).

The added complexity of a KAFO powered by antagonistic pairs of artificial pneumatic muscles could limit its performance. First, actuator force-length properties [5] and smaller moment arms could lead to reduced torque from artificial pneumatic muscles acting at the knee. Second, antagonistic artificial muscle pairs under proportional myoelectric control could result in co-activation reducing the net assistance torque. We evaluated the performance of our powered KAFO in the context of two key questions: (1) Would the KAFO deliver assistance torque at the knee joint with timing and magnitude similar to that of the biological muscle-tendon moments during normal walking without the orthosis? (2) Would using leg extensor muscle EMG signals to inhibit flexor artificial pneumatic muscles lead to improved gait kinematics than direct proportional myoelectric control that includes co-activation of antagonistic artificial muscles?

To address these questions we compared overground walking trials without the orthosis (baseline), with the KAFO unpowered, and with the KAFO powered under two distinct proportional myoelecric control modes. The first control mode allowed co-activation of artificial extensor and flexor muscles (at both joints) (PM – direct proportional myoelectric). The second, modeled after reciprocal inhibition observed in humans [22], prevented co-activation by inhibiting flexor activation when the antagonist extensor was active (PMFI – proportional myoelectric control with flexor inhibition).

## Methods

### Subjects

We tested three healthy male subjects (body mass 91.9 ± 17.2 kg; height 187.0 ± 3.4 cm, mean ± s.d.). Each subject read and signed a consent form prepared according the Declaration of Helsinki and the protocol was approved by the University of Michigan Institutional Review Board for human subject research.

### Orthosis hardware

**Figure 1:**
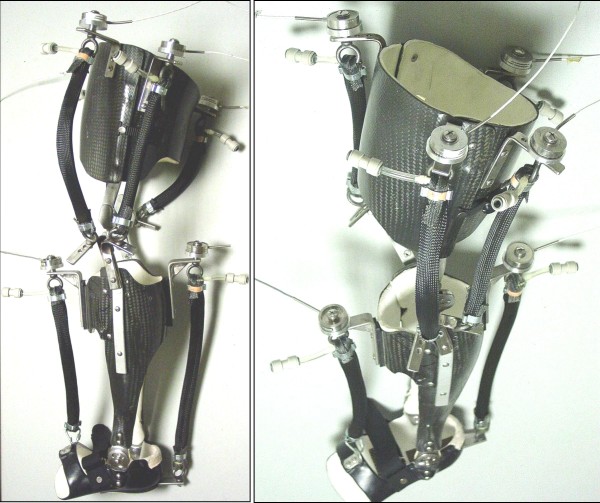
**University of Michigan Knee-Ankle-Foot Orthosis (KAFO)**. Two pictures of the unilateral (left leg) knee-ankle-foot orthosis (KAFO) with artificial pneumatic muscles display the thigh and shank sections made from carbon-fiber and the foot section made from polypropylene. The orthoses were custom molded from a cast unique to each subject. Hinge joints at the ankle and knee allowed free motion in the sagittal plane. We used steel brackets to attach two artificial pneumatic muscles (a plantar flexor and a dorsiflexor) around the ankle and four around the knee (two extensors and two flexors). Each artificial pneumatic muscle had a compression load transducer mounted in series on the proximal steel bracket attachment and a release valve for quick connection to the pressurized air source. A special shoe was worn over the foot section during walking trials.

We constructed a single (left leg only), custom-fit knee-ankle-foot orthosis for each subject (Figure 1). The knee-ankle-foot orthosis (KAFO) concept was extended from our previously described ankle-foot orthosis (AFO) designs [4,5,23]. The lightweight orthosis (mass 2.9 ± 1.3 kg) consisted of a polypropylene foot section, a carbon fiber shank and a carbon fiber thigh. Hinge joints allowed free ankle dorsi-plantar flexion and knee flexion-extension.

We attached six artificial pneumatic muscles to each orthosis. The pneumatic muscles were an ankle dorsiflexor, an ankle plantar flexor, two knee extensors, and two knee flexors. Each artificial pneumatic muscle was attached to the orthosis with stainless steel brackets. We positioned each bracket in order to achieve the largest possible artificial muscle moment arm while maintaining the normal joint range of motion. Additional details on specifications for the orthoses and their components can be found in Table 1.

**Table 1:** Knee-ankle-foot orthosis specifications

**Orthosis**	Component Mass (g)		**Artificial Muscles**	Muscle Length (cm)		Moment Arm Length (cm)	
				
	Mean	*SD*		Mean	*SD*	Mean	*SD*
				
**Artificial Muscle**	128	*7*	**Plantarflexor**	47.5	*2.3*	10.3	*1.0*
**Load Cell**	94	*0*	**Dorsiflexor**	38.5	*4.4*	11.3	*1.6*
**Blow Valve**	152	*0*	**Medial Knee Extensor**	34.0	*5.2*	3.2	*0.6*
**Thigh**	1089	*90*	**Lateral Knee Extensor**	34.0	*1.0*	2.8	*0.8*
**Shank**	1408	*89*	**Medial Knee Flexor**	29.0	*3.0*	4.0	*1.0*
**Foot**	388	*34*	**Lateral Knee Flexor**	31.0	*2.0*	3.2	*1.0*
	
**Total**	2884	*134*					

We used eight (4 for the ankle pneumatic muscles, 4 for the knee pneumatic muscles) parallel proportional pressure regulators (valve PPC0445A-ACA-OAGABA09 and solenoid 45A-L00_DGFK-1BA, MAC Valves, Inc. Wixom, MI) to supply compressed air to each artificial muscle via nylon tubing (0–6.2 bar). Analog-controlled solenoid valves in parallel with the air supply tubing improved exhaust dynamics (35A-AAA-0DAJ-2KJ, MAC Valves, Inc., Wixom, MI).

### Artificial pneumatic muscle control

We implemented a physiologically-inspired controller that incorporated the user's own surface electromyography to dictate the timing and magnitude of artificial muscle forces (i.e. proportional myoelectric control). We chose to control each artificial pneumatic muscle with an electromyography signal generated by a biological muscle with analogous mechanical action. That is, artificial extensors were controlled by biological extensors and artificial flexors were controlled by biological flexors. More specifically, at the ankle we used tibialis anterior to control the artificial dorsiflexor and soleus to control the artificial plantar flexor. At the knee, we used vastus lateralis to control the two artificial knee extensors and medial hamstrings to control the two artificial knee flexors.

We programmed two proportional myoelectric control modes using a real-time computer interface (dSPACE Inc., Northville, MI; 1000 Hz sampling). The first allowed co-activation of artificial extensor and flexor muscles (proportional myoelectric, PM) and the second prevented co-activation by inhibiting flexor activation when the antagonist extensor was active (proportional myoelectric with flexor inhibition, PMFI). In both cases we amplified, high pass filtered (f_c _= 50 Hz), full-wave rectified, low pass filtered (f_c _= 10 Hz) and then applied a threshold and gain to convert the raw voltage recorded from surface electrodes to the voltage commanding the pneumatic hardware. The time between the control signal onset and initial rise of artificial muscle tension (~50 ms) of the device was comparable to response times of human muscles [23].

### Protocol

At the start of the session, subjects walked for 10 minutes on a motorized treadmill at 1.25 m/s wearing the KAFO unpowered (i.e. with artificial muscles turned off). During the unpowered treadmill walking bout we tuned the proportional myoelectric controller gains and thresholds for each artificial muscle. The same gains and thresholds were used in the PM and PMFI control modes. For the artificial plantar flexor we used G = 0.17 ± 0.06 V/μV, Th = 18.7 ± 5.5 μV; dorsiflexor G = 0.22 ± 0.03 V/μV, Th = 24.7 ± 5.0 μV; knee extensors G = 0.37 ± 0.07 V/μV, Th = 7.3 ± 6.8 uV; and knee flexors G = 0.30 ± 0.07 V/μV, Th = 15.0 ± 8.9 μV. We chose the threshold to eliminate background noise and the gain to get a saturated control signal (10 V) at peak for at least five consecutive steps.

Subjects then completed five overground walking trials at 1.25 m/s with the orthosis in three different conditions: (1) unpowered, (2) powered under proportional myoelectric control (PM) and (3) powered under proportional myoelectric control with flexor inhibition (PMFI) (i.e. a total of 15 overground trials). Following the orthosis trials, subjects completed five more overground trials at 1.25 m/s without wearing the orthosis in order to establish a baseline for comparisons.

### Data collection and analysis

We collected joint kinematics, ground reaction forces, surface electromyography and artificial muscle force data during over ground walking trials at 1.25 m/s. To ensure that trials were within ± 0.05 m/s of the target speed, we used infrared timers triggered at beginning and end of the ~12 meter walkway. For all reported time series data, we first formed profiles for a normalized stride cycle using foot-switches placed in the shoe (1200 Hz, B&L Engineering, Tustin, CA, USA) to mark consecutive left heel strikes (0% and 100% of the stride). For each subject, we averaged the stride normal data from each of the five trials in each condition (Without, Unpowered, PM, PMFI) to get stride cycle average time-series profiles. For each condition we averaged across subjects to form the mean stride cycle average time-series traces reported in figures.

#### Joint Kinematics

To compute ankle, knee and hip joint angles we used an 8-camera video system (frame rate 120 Hz, Motion Analysis Corporation, Santa Rosa, CA, USA) to record the positions of twenty-nine reflective markers on the subjects' pelvis and lower limbs. We used custom software (Visual 3D, C-Motion, Rockville, MD, USA) to smooth the raw marker data (4^th^-order low pass Butterworth, f_c _= 6 Hz) and calculate joint angles (relative to neutral standing posture) and angular velocities.

#### Ground Reaction Forces and Joint Kinetics

We used a single force platform (sampling rate 1200 Hz, Advanced Mechanical Technology Inc., Watertown, MA, USA) to record the ground reaction force under the left foot. Combining ground reaction force data and joint kinematic data, we used inverse dynamics to calculate ankle, knee and hip joint net muscle-tendon moments and powers over the stride (Visual 3D software, C-Motion, Rockville, MD, USA). We used standard regression equations to estimate subjects' anthropometry [24] and adjusted foot and shank parameters to account for added orthosis mass and inertia. We divided moments (N-m) by subject plus orthosis mass to make them mass-specific (N-m/kg).

We quantified the mass-specific mechanical work delivered by the ankle and knee moments for one leg over the stride. First we integrated the positive and negative portions of the ankle and knee mechanical power curves separately, then summed the portions and finally divided by the subject plus orthosis mass.

#### Orthosis Mechanics

We used single-axis compression load transducers (1200 Hz, Omega Engineering, Stamford, CT, USA) to record the forces produced by the artificial pneumatic muscles during orthosis walking trials (Figure 1). We measured the artificial muscle moment arms with the ankle and knee joints in the neutral position during upright standing posture (Table 1). We multiplied moment arm length and smoothed artificial muscle force data (4^th^-order low pass Butterworth, f_c _= 6 Hz) to compute orthosis ankle and knee torques. To determine the mechanical power delivered by the orthosis, we multiplied the orthosis torques and joint angular velocities. We divided torques (N-m) by subject plus orthosis mass to make them mass-specific (N-m/kg). We computed the mass-specific positive (and negative) mechanical work delivered by the orthosis ankle and knee torques over the stride in the same manner as was done for the mechanical work performed by the joint net muscle-tendon moments.

#### Electromyography

We recorded lower-limb surface electromyography (EMG) (1200 Hz, Konigsberg Instruments, Inc., Pasadena, CA, USA) from the left soleus (Sol), tibialis anterior (TA), vastus lateralis (VL) and medial hamstrings (MH) using bipolar electrodes (inter-electrode distance 3.5 cm) centered over the belly of the muscle along its long axis. We performed simple functional tests (i.e. joint flexion or extension against resistance) to verify that our electrode placements gave appropriate signals for each muscle. EMG amplifier bandwidth filter was 12.5 Hz – 920 Hz. We placed electrodes to minimize cross-talk and taped them down to minimize movement artifact. We high-pass filtered (4^th^-order Butterworth, f_c _= 50 Hz), rectified and low-pass filtered (4^th^-order Butterworth, f_c _= 10 Hz) each of the EMG signals (i.e. linear envelope).

#### Statistical Analyses

To assess the effect of orthosis control mode (PM or PMFI) on orthosis mechanical performance (joint kinematics and joint kinetics) we performed Pearson product moment correlations (i.e. r-values). For joint kinematics, we correlated the mean stride cycle average time-series for ankle, knee and hip joint angles for PM-to-Without and PMFI-to-Without pairings. Similarly, for orthosis kinetics, we correlated the mean stride cycle average time-series for the orthosis ankle and knee torque and power curves during the powered conditions (PM and PMFI) to the ankle and knee joint net muscle-tendon moment and power curves during walking without the orthosis (Without) (i.e. PM-Without and PMFI-Without pairings).

We used JMP statistical software (SAS Institute, Inc. Cary, NC, USA) to perform repeated measures analysis of variance tests (ANOVAs) on (1) the r-values from the above described Pearson product moment correlations, and (2) the positive and negative mechanical work values calculated from ankle and knee joint mechanical power curves (without) and ankle and knee orthosis power curves (PM and PMFI) (two-way tests: subject, mode). When we found a significant effect (p < 0.05) we used post-hoc Tukey Honestly Significant Difference (THSD) tests to determine specific differences between means. We performed statistical power analyses for each test (see Tables 2 and 3).

**Table 2:** Moment, Power and Angle Correlations to Without Orthosis Walking

	**Pearson r-value**					
			
	**PM**		**PMFI**		**ANOVA p-value; ** **Power**	**THSD**
				
	Mean	*SE*	Mean	*SE*		
				
**Orthosis Ankle** **Torque**	0.85	*0.05*	0.76	*0.11*	**p = 0.28** ***P = 0.14***	
**Orthosis Ankle** **Power**	0.53	*0.11*	0.72	*0.07*	*p = 0.04 ***P = 0.70***	**PMFI > PM**
**Orthosis Knee** **Torque**	-0.01	*0.21*	0.55	*0.04*	**p = 0.09** ***P = 0.42***	
**Orthosis Knee** **Power**	-0.03	*0.06*	0.17	*0.11*	**p = 0.33** ***P = 0.12***	
**Ankle Angle**	0.49	*0.13*	0.74	*0.04*	*p = 0.05 ***P = 0.80***	**PMFI > PM**
**Knee Angle**	0.90	*0.03*	0.95	*0.03*	**p = 0.17** ***P = 0.24***	
**Hip Angle**	0.98	*0.01*	0.98	*0.00*	**p = 0.71** ***P = 0.06***	

Values are Mean ± *Standard Error *for n = 3 subjects.

*Indicates a p-value of less than 0.05 showing significant differences between conditions.

*Statistical power, P*, is reported under the p-value.

Tukey Honestly Significant Difference (THSD) results are reported for metrics with significance.

**PM **= proportional myoelectriccontrol **PMFI **= proportional myoelectric control with flexor inhibition

**Table 3:** Mechanical Work Summary

		**Work (J/kg)**							
				
		**Without**		**Orthosis PM**		**Orthosis PMFI**		**ANOVA p-value;** **Power**	**THSD**
						
		Mean	*SE*	Mean	*SE*	Mean	*SE*		
						
**ANKLE**	**Pos.**	0.21	*0.03*	0.18	*0.03*	0.21	*0.02*	**p = 0.52** ***P = 0.11***	
	**Neg.**	0.25	*0.03*	0.10	*0.02*	0.11	*0.02*	*p = 0.03 ***P = 0.76***	**PM < WO** **PMFI < WO**
									
**KNEE**	**Pos.**	0.05	*0.02*	0.06	*0.02*	0.09	*0.03*	**p = 0.65** ***P = 0.09***	
	**Neg.**	0.31	*0.02*	0.04	*0.02*	0.06	*0.03*	*p = 0.003 ***P = 0.99***	**PM < WO** **PMFI < WO**

Values are Mean ± *Standard Error *for n = 3 subjects.

*Indicates a p-value of less than 0.05 showing significant differences between conditions.

*Statistical power, P*, is reported under the p-value.

Tukey Honestly Significant Difference (THSD) results are reported for metrics with significance.

**PM **= proportional myoelectric control **PMFI **= proportional myoelectric control with flexor inhibition

## Results

### Without orthosis versus unpowered orthosis

**Figure 2:**
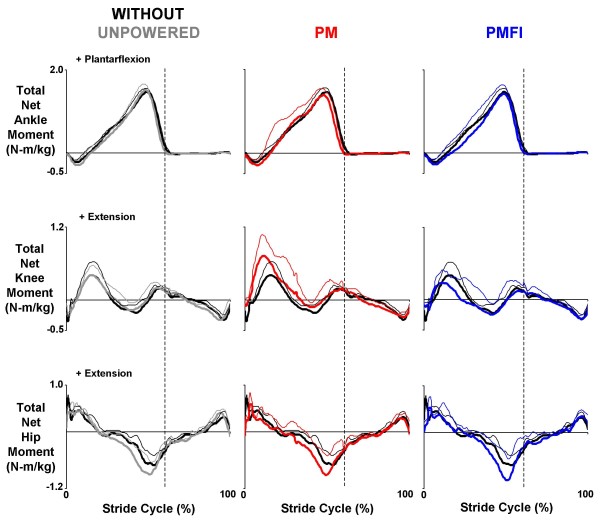
**Ankle, knee and hip total net joint moments**. Mean (thick lines) + 1 standard deviation (thin lines) stride cycle average (0%-left heel strike to 100%-left heel strike) total net joint moments for the ankle, knee and hip. Plotted values were normalized by subject mass (N-m/kg). The total moment was measured externally and included contributions from biological muscle-tendons and orthosis artificial muscles (except for the hip in all conditions and for the ankle and knee in the without orthosis condition). Data across rows (from left to right) were for walking at 1.25 m/s overground with the orthosis unpowered (Unpowered, gray), powered under proportional myoelectric control (PM, red) and powered under proportional myoelecric control with flexor inhibition (PMFI, blue). In each panel, traces are compared to normal walking without wearing the orthosis (Without, black). Dotted vertical lines mark the stance-swing transition at ~60% of the stride cycle. Positive values indicate ankle plantar flexor, knee extensor and hip extensor moments.

**Figure 3:**
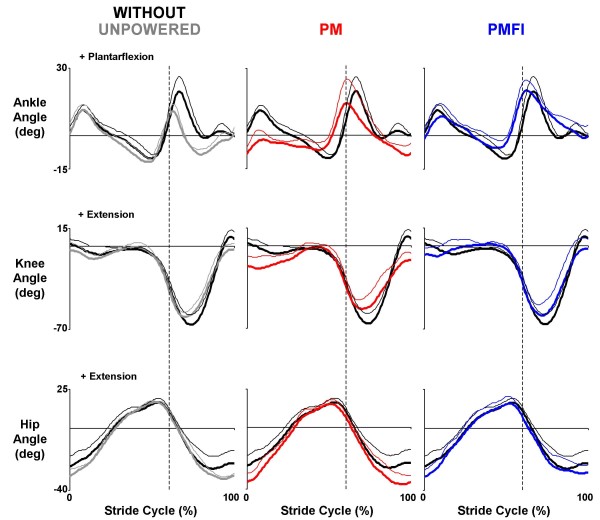
**Ankle, knee and hip joint angles**. Three subject mean (thick lines) + 1 SD (thin lines) stride cycle average (0%-left heel strike to 100%-left heel strike) joint angles (deg) for the ankle, knee and hip. Data across rows (from left to right) are for walking at 1.25 m/s overground with the orthosis unpowered (Unpowered, gray), powered under proportional myoelectric control (PM, red) and powered under proportional myoelecric control with flexor inhibition (PMFI, blue). In each panel, traces are compared to normal walking without wearing the orthosis (Without, black). Dotted vertical lines mark the stance-swing transition at ~60% of the stride cycle. Angles are measured with reference to quiet standing posture. Positive angles indicate ankle plantarflexion, knee extension and hip extension.

**Figure 4:**
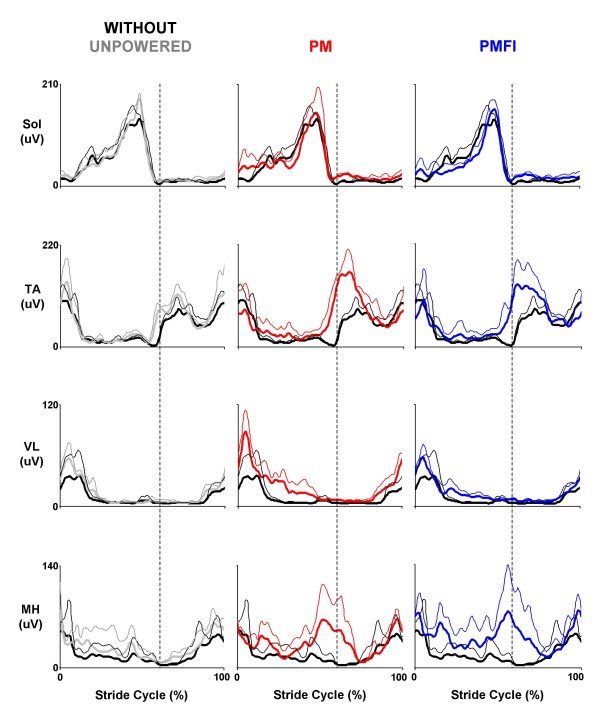
**Ankle and knee muscle surface electromyography**. Three subject mean (thick lines) + 1 SD (thin lines) stride cycle average (0%-left heel strike to 100%-left heel strike) electromyography amplitudes (uV) for the knee-ankle-foot orthosis control muscles at the ankle (Sol – soleus and TA – tibialis anterior) and the knee (VL – vastus lateralis and MH – medial hamstrings). Data across rows (from left to right) are for walking at 1.25 m/s overground with the orthosis unpowered (Unpowered, gray), powered under proportional myoelectric control (PM, red) and powered under proportional myoelecric control with flexor inhibition (PMFI, blue). In each panel, traces are compared to normal walking without wearing the orthosis (Without, black). Dotted vertical lines mark the stance-swing transition at ~60% of the stride cycle.

All three subjects were able to walk comfortably while wearing the knee-ankle-foot orthosis (KAFO) with artificial pneumatic muscles turned off. Kinetic (net joint muscle-tendon moments and powers), kinematic (joint angles), and surface electromyography profiles for walking with the orthosis unpowered were similar to those walking without the orthosis (Figures 2, 3, 4).

### Orthosis ankle joint performance: PM versus PMFI

**Figure 5:**
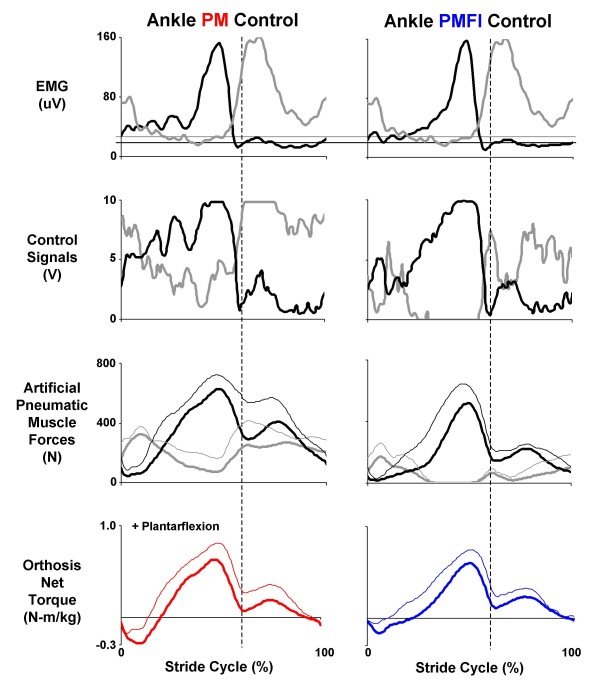
**Orthosis ankle joint control**. Data are mean (solid lines) + 1 SD (thin lines where reported) for three subjects walking at 1.25 m/s with the knee-ankle-foot orthosis powered in two control modes: direct proportional myoelectric control (PM, left column) and proportional myoelectric control with flexor inhibition (PMFI, right column). Each column (from top to bottom) shows: surface electromyography (μV) from the users' soleus (black) and tibialis anterior (grey); processed pneumatic control signals (V) for the artificial plantarflexor (black) and dorsifelxor (grey); the forces (N) generated by the antagonistic artificial muscles (plantar flexor in black and dorsiflexor in grey); and the resulting net artificial muscle torque (N-m/kg) delivered at the ankle joint (PM, left column in red and PMFI, right column in blue). Plantar flexor torque is positive. All data are plotted over a stride cycle from left heel strike (0%) to left heel strike (100%). Dashed vertical lines at 60% of the stride mark the stance-swing transition. Horizontal lines on the electromyography traces indicate the threshold setting (μV) above which a proportional control signal is generated (plantar flexor in black and dorsiflexor in grey).

Soleus and tibialis anterior electromyography (EMG) patterns were nearly identical for the two proportional myoelectric control conditions (PM vs. PMFI) but the control signals generated were markedly different (Figures 4, 5). Due to the flexor inhibition algorithm, the control signal voltage was much lower for the artificial dorsiflexor during the stance phase (~3 V versus 0 V) in the PMFI versus PM control mode.

Artificial muscle force patterns reflected the differences in control signals between the proportional myoelectric control modes. With direct proportional myoelectric control (PM), the artificial plantar flexor force peaked late in stance at 645 ± 57 N (mean ± SEM). With the flexor inhibition algorithm (PMFI), the peak artificial plantar flexor force was only 533 ± 71 N. Artificial dorsiflexor forces followed a similar trend, peaking early in stance at 388 ± 27 N during powered walking under PM control but reaching a peak of only 196 ± 60 N during PMFI (Figure 5).

**Figure 6:**
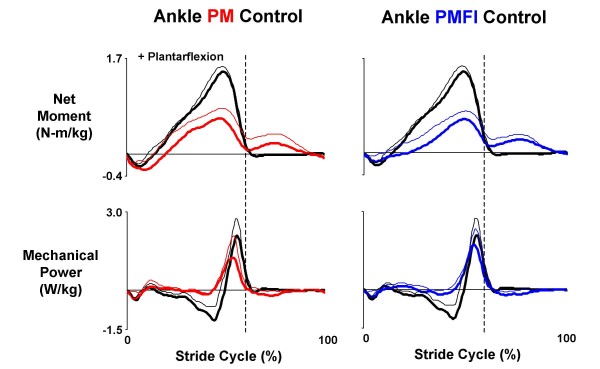
**Orthosis ankle joint kinetics**. Data traces are mean (solid lines) + 1 SD (thin lines) from the left leg of three subjects who walked overground at 1.25 m/s with a knee-ankle-foot orthosis powered in two control modes. In the left column, data from powered trials with direct proportional myoelectric control (PM, red) for net torque (N-m/kg) (top) generated by the ankle joint artificial muscles and the resulting mechanical power (W/kg) (bottom) is compared with the net ankle muscle-tendon moment (N-m/kg) and mechanical power (W/kg) recorded during walking without the orthosis (black). The right column shows similar data for powered trials using proportional myoelectric control with a flexor inhibition algorithm (PMFI, blue). Stride cycles begin (0%) and end (100%) at left heel strike. Dotted vertical lines mark the stance-swing transition at ~60% of the stride cycle. Plantar flexor torque/moment is positive. Positive mechanical power indicates energy generation and negative mechanical power indicates energy absorption.

The flexor inhibition controller (PMFI) reduced co-activation of the antagonist artificial plantar flexor and dorsiflexors compared to direct proportional myoelectric control (PM), but it also reduced net torque magnitudes (Figure 5). In PM control mode, the orthosis delivered 0.67 ± 0.09 N-m/kg peak plantar flexor torque near the end of the stance phase and -0.31 ± 0.08 N-m/kg peak dorsiflexor torque early in the stance phase (Figure 5). These values were ~46% and ~129% of peak biological plantar flexor and dorsiflexor net muscle-tendon moments from walking without the orthosis (Figure 6). In PMFI control mode, peak ankle orthosis torques were reduced to 0.62 ± 0.09 N-m/kg peak plantar flexor and -0.20 ± 0.09 N-m/kg peak dorsiflexor (Figure 5). These were 42% and 83% of peak biological plantar flexor and dorsiflexor net muscle-tendon moments (Figure 6). Despite reductions in peak torque magnitudes for PMFI versus PM control, the orthosis torque patterns during PMFI and PM control were equally similar to the ankle moment during walking without the orthosis. The Pearson product moment correlation (r-value) for ankle torque was not significantly different for PMFI-Without (0.76 ± 0.11) versus PM-Without (0.85 ± 0.05) (p = 0.28) (Table 2).

The flexor inhibition algorithm (PMFI) resulted in greater mechanical power generation at the orthosis ankle joint compared to direct proportional myoelectric control (PM). Biological ankle muscle-tendon positive mechanical power peaked at 2.19 ± 0.38 W/kg during normal walking at 1.25 m/s without the orthosis. During powered walking under direct PM control, the orthosis peak positive power was 1.45 ± 0.35 W/kg (Figure 6). With PMFI control, the orthosis peak positive power was 1.88 ± 0.28 W/kg, a 30% increase over PM control. Furthermore, the orthosis ankle positive mechanical work also tended higher during powered walking with PMFI control (0.21 ± 0.02 J/kg) versus PM control (0.18 ± 0.03 J/kg) (Table 3). The ankle mechanical power Pearson product moment correlation for PMFI-Without (0.72 ± 0.07) was significantly higher than the correlation for PM-Without (0.53 ± 0.11) (p = 0.04) (Table 2).

The ankle joint artificial muscles did a poor job absorbing mechanical energy under both proportional myoelectric control modes. Except for early in stance, when net orthosis dorsiflexor torque absorbed energy to prevent foot drop, the ankle orthosis performed very little negative mechanical work (Figure 6). In both control modes (PM and PMFI), the orthosis performed ~40% less negative work than the biological ankle muscle-tendon moment during walking without the orthosis (p = 0.03) (Table 3).

The total net ankle joint moment (net orthosis ankle torque + biological ankle net muscle-tendon moment) was qualitatively similar between the powered walking conditions (PM versus PMFI) and walking without the orthosis (Figure 2).

Ankle joint kinematics during walking without the orthosis were much more similar to ankle joint kinematics during powered walking with flexor inhibition (PMFI) compared to powered walking without flexor inhibition (PM). With direct proportional myoelectric control (PM), the ankle joint was more dorsiflexed both early in stance and late in swing when compared to normal walking without the orthosis. In contrast, the ankle angle profile during powered walking under PMFI control was very similar to walking without the orthosis (Without) (Figure 3). The Pearson product moment correlation for ankle angle was significantly higher for PMFI-Without (0.74 ± 0.04) versus PM-Without (0.49 ± 0.13) time-series comparisons (p = 0.05) (Table 2).

When compared to normal walking without the orthosis (Without), ankle muscle electromyography (soleus and tibilais anterior) patterns were altered during powered walking under both proportional myoelectric control modes. During powered walking with direct proportional myoelectric control (PM), soleus muscle activity was slightly greater than normal early in stance and tibialis anterior activity was markedly higher than normal in early swing (Figure 4). Although perhaps slightly attenuated, there were similar increases in muscle activity during powered walking with flexor inhibition (PMFI) (Figure 4).

### Orthosis knee joint performance: PM versus PMFI

**Figure 7:**
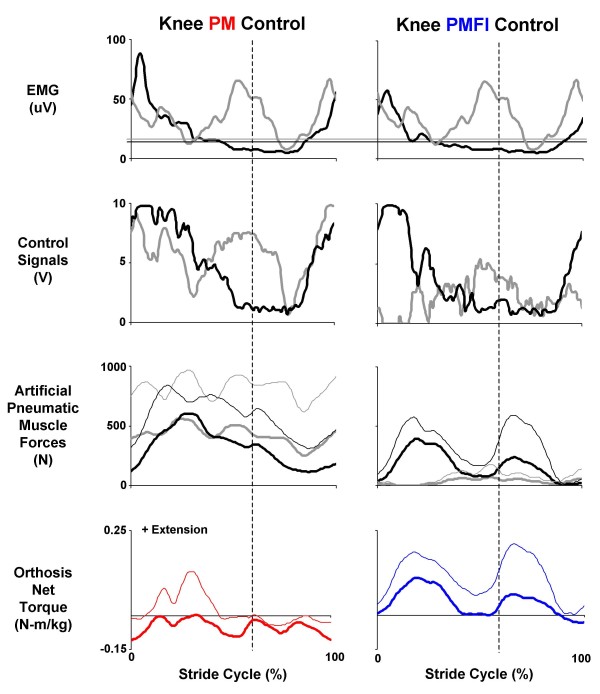
**Orthosis knee joint control**. Data are mean (solid lines) + 1 SD (thin lines where reported) for three subjects walking at 1.25 m/s with the knee-ankle-foot orthosis powered in two control modes: direct proportional myoelectric control (PM, left column) and proportional myoelectric control with flexor inhibition (PMFI, right column). Each column (from top to bottom) shows: surface electromyography (μV) from the users' vastus lateralis (black) and medial hamstrings (grey); processed pneumatic control signals (V) for the artificial knee extensor (black) and knee flexor (grey); the forces (N) generated by the antagonistic artificial muscles (extensors in black and flexors in grey); and the resulting net artificial muscle torque (N-m) delivered at the knee joint (PM, left column in red and PMFI, right column in blue). Knee extensor torque is positive. All data are plotted over a stride cycle from left heel strike (0%) to left heel strike (100%). Dashed vertical lines at 60% of the stride mark the stance-swing transition. Horizontal lines on the electromyography traces indicate the threshold setting (μV) above which a proportional control signal is generated (knee extensor in black and knee flexor in grey).

Knee artificial muscle co-activation was nearly eliminated with the flexor inhibition algorithm (PMFI) compared to direct proportional myoelectric control (PM). During powered walking in PM control, the artificial knee extensors and flexors were co-activated over the entire stride. The two artificial knee extensors combined to produce peak forces in mid-stance of 629 ± 72 N. The two artificial flexors combined to produce a nearly constant force over the stride, peaking at 472 ± 147 N. During powered walking in PMFI control, both peak knee extensor (494 ± 79 N) and peak knee flexor (162 ± 18 N) forces were reduced (Figure 7).

**Figure 8:**
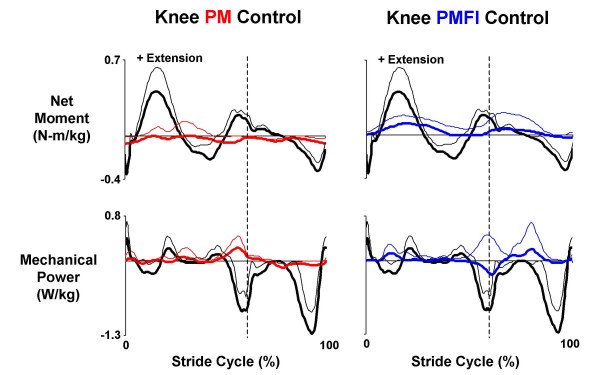
**Orthosis knee joint kinetics**. Data traces are mean (solid lines) + 1 SD (thin lines) from the left leg of three subjects who walked overground at 1.25 m/s with a knee-ankle-foot orthosis powered in two control modes. In the left column, data from powered trials with direct proportional myoelectric control (PM, red) for net torque (N-m/kg) (top) generated by knee joint artificial muscles and the resulting mechanical power (W/kg) (bottom) is compared with the net ankle muscle-tendon moment (N-m/kg) and mechanical power (W/kg) recorded during walking without the orthosis (black). The right column shows similar data for powered trials using proportional myoelectric control with a flexor inhibition algorithm (PMFI, blue). Stride cycles begin (0%) and end (100%) at left heel strike. Dotted vertical lines mark the stance-swing transition at ~60% of the stride cycle. Knee extensor torque/moment is positive. Positive mechanical power indicates energy generation and negative mechanical power indicates energy absorption.

The orthosis knee net joint torque was drastically different during powered walking with PM versus PMFI control. During walking in direct proportional myoelectric control (PM), the knee artificial flexors and extensors co-activated, stiffening the joint, and delivered a small net flexor torque over the entire stride (Figure 7). The knee orthosis torque pattern did not coincide (in magnitude or timing) with the biological knee net muscle-tendon moments (Figure 8). For powered walking under PM control mode, the powered knee peak extensor torque was 0.10 ± 0.03 N-m/kg and peak flexor torque was -0.13 ± 0.02 N-m/kg. These reached only 22% of peak biological knee extensor (0.45 ± 0.10 N-m/kg) and 33% of peak biological knee flexor (-0.39 ± 0.03 N-m/kg) net muscle-tendon moments from walking without the orthosis (Figure 8). In contrast, during PMFI, the peak knee orthosis extensor torque during powered walking was 50% higher (0.15 ± 0.04 N-m/kg) than during powered walking under PM control. Furthermore, the timing of the orthosis knee torque during PMFI was more similar to normal walking when compared to PM. The Pearson product moment correlation for knee torque was greater for PMFI-Without (0.55 ± 0.04) compared to PM-Without (-0.01 ± 0.21) (p = 0.09) (Table 2). Although the flexor inhibition controller had better orthosis extensor torque timing and magnitude, the knee orthosis peak flexor torque during PMFI was smaller (-0.06 ± 0.03) than during PM. The knee orthosis peak flexor torque during PMFI was only 15% of biological knee flexor peak net muscle-tendon moment during walking without the orthosis (Figure 7).

Total net knee joint moment (net orthosis knee torque + biological knee net muscle-tendon moment) was qualitatively more similar to walking without the orthosis with PMFI versus PM control (Figure 2).

Mechanical power delivered by the orthosis knee joint was greater with the flexor inhibition control algorithm (PMFI) compared to the direct proportional myoelectric control (PM). The Pearson product moment correlation for knee mechanical power was higher for PMFI-Without (0.17 ± 0.11) versus PM-Without (-0.03 ± 0.06) (p = 0.33) (Table 2). However, the orthosis knee was poor at absorbing mechanical energy for both powered conditions. The orthosis knee artificial muscles absorbed significantly less mechanical energy than biological knee muscle-tendons during normal walking (p = 0.003) (Figure 8) (Table 3). During powered walking under PM, the knee net orthosis torque performed -0.04 ± 0.02 J/kg negative work versus -0.06 ± 0.03 J/kg during walking under PMFI control.

The flexor inhibition controller (PMFI) produced knee kinematics that were similar to walking without the orthosis (Figure 3). With PM control, the knee was more flexed during stance and less flexed during swing than normal walking. Increased knee flexion was absent during powered walking under PMFI control. The Pearson product moment correlation for knee angle was greater for PMFI-Without (0.95 ± 0.03) compared to PM-Without (0.90 ± 0.03), but not significantly so (p = 0.17) (Table 2).

Knee muscle electromyography was different in both powered walking conditions (PM and PMFI) when compared to normal walking without the orthosis (Without). For vastus lateralis, during powered walking with direct proportional myoelectric control (PM), muscle activity was greater than normal throughout stance and late in swing. For medial hamstrings, activity was markedly higher than during the stance-swing transition (Figure 4). Knee muscle activity patterns were similar for powered walking under direct PM and the flexor inhibition (PMFI) control (Figure 4).

### Hip joint kinetics and kinematics: PM versus PMFI

Total hip joint net muscle moments (Figure 2) and hip joint kinematics (Figure 3, Table 2) during powered walking were similar to normal walking without the orthosis (Without). This was true for both proportional myoelectric control modes (PM and PMFI).

## Discussion

The addition of a flexor inhibition algorithm (PMFI) to the standard proportional myoelectric controller (PM) allowed naïve users of the powered knee-ankle-foot orthosis to walk with their normal gait. The flexor inhibition algorithm reduced artificial pneumatic muscle co-activation and produced joint kinematics and joint kinetics at both the knee and ankle that were similar to walking without the orthosis (Figures 2, 3, 6 and 8). A modified version of the controller that would more closely mimic human neurophysiology might include inhibition of artificial extensor muscles from leg flexor electromyography during swing [22,25]. This modification could potentially result in gait dynamics even more similar to normal unassisted walking.

In general, proportional myoelectric control has specific advantages and disadvantages for control of lower limb robotic orthoses compared to other control approaches. The advantages are: 1) it provides an effective way to scale the magnitude of the orthosis mechanical assistance due to its physiological nature [3,26], 2) it appears to lead to a greater reduction in biological muscle recruitment compared to kinematic based control algorithms [27], and 3) it easily allows the nervous system to adapt orthosis control for novel motor tasks. The disadvantages are: 1) it can be difficult to obtain a reliable and consistent myoelectric signal due to the surface electrode interface [26], 2) choosing the appropriate threshold and gain requires tuning [28], and 3) the complexity of the musculoskeletal system includes many synergistic muscles that are not all easily accessible with surface electromyography [29]. Development of small wireless intramuscular electrodes and adaptive control algorithms could substantially attenuate these disadvantages in the near future [30,31].

A common question about proportional myoelectric control as a means for operating powered orthoses for neurological rehabilitation is "why use a neural signal that is weak and disordered to guide robotic assistance?". The answer to that question is based on motor learning theory. Motor learning is directly related to how our nervous system detects motor error. When inappropriate electrical signals are sent to the muscles of a limb, the proprioceptive (and visual) feedback about the limb's performance allows the nervous system to determine if there was error in the performance. Performance error is the major impetus for dynamic motor adaptation [32,33]. A neurologically impaired human that sends a weak and noisy electrical signal to their muscles will have difficulty deducing when the signal was appropriate and when it was inappropriate. This is especially true if the proprioceptive feedback is also weak and noisy. Proportional myoelectric control of artificial pneumatic muscles on a robotic orthosis provides a way to amplify the consequences of the electrical signal sent to the biological muscles. This makes it easier for the nervous system to detect performance error and alter the subsequent electrical commands to the muscles. This approach could be considered similar in a way to the error amplification control algorithm suggested by Patton and colleagues for robotic rehabilitation devices [34,35].

The current orthosis design provided less mechanical assistance about the knee than about the ankle. This is largely due to the trade off in torque and range of motion that occurs with artificial pneumatic muscles. The knee joint undergoes about twice the angular displacement of the ankle joint during human walking (Figure 3). To provide for the greater range of motion at the knee, the moment arms of the artificial knee muscles were only about one third of the moment arms for the artificial ankle muscles. Artificial pneumatic muscles can only shorten about one third of their resting length and cannot be stretched substantially beyond their resting length without breaking. Using greater moment arms for the knee artificial muscles would have required greater artificial muscle displacements than what was possible. Another factor limiting energy transmission from artificial muscles to the user's joint is compliance in the orthosis carbon fiber shell [5]. Future designs could include stiffer shell materials, larger circumference artificial pneumatic muscles, cams, gears, Bowden cable transmission [36,37] or different actuators (e.g. pneumatic cylinders) to alleviate limitations in knee torque production.

Consistent with our previous results using a powered ankle-foot orthosis [28,38], the artificial muscles performed more positive mechanical work than negative mechanical work during the gait cycle (Table 3). This is likely related to the inherent mechanical characteristics of artificial pneumatic muscles. Although artificial muscles can perform negative work, their force-length properties result in a steep linear increase in force as they lengthen (assuming constant activation) [39-41]. The large increase in artificial muscle force during stretch makes it difficult to perform extended negative mechanical work against inertial loads like human body mass. It would be possible to decrease the activation amplitude of an artificial pneumatic muscle as it lengthens to keep force relatively stable, but this does not seem easy for the human nervous system to do with proportional myoelectric control [28,38]. Thus, for robotic orthoses intended to perform primarily negative mechanical work (e.g. at the knee joint), it might be preferable to use actuators that can provide variable damping [21,42].

Powered knee-ankle-foot orthoses have promising clinical and basic science applications. We have successfully applied our ankle device and its control architecture to assist individuals with incomplete spinal cord injury during locomotor training [43] and to study neural adaptation [27-29,44] and metabolic energy consumption in neurally intact human walkers [38,45,46]. Future studies will aim to extend these research paradigms to the knee and ultimately, the hip.

## Competing interests

The authors declare that they have no competing interests.

## Authors' contributions

GSS recruited subjects, managed data collections, completed data analysis and drafted the manuscript. DPF conceived of the study, provided guidance on experimental design and helped edit the manuscript. All authors read and approved the final manuscript.
